# DEVELOPMENT OF A MEASURE OF ALF RESIDENTS’ ACTIVITY PREFERENCES AND REINFORCERS

**DOI:** 10.1093/geroni/igae098.3164

**Published:** 2024-12-31

**Authors:** Nicole Praska, Daniel Houlihan, Jeff Buchanan, Rachel Weiskittle

**Affiliations:** University of Colorado, Colorado Springs, Colorado Springs, Colorado, United States; Minnesota State University Mankato, Mankato, Minnesota, United States; Minnesota State University Mankato, Mankato, Minnesota, United States; University of Colorado, Colorado Springs, Colorado Springs, Colorado, United States

## Abstract

The population of individuals aged 65 and older has grown steadily since the 1990s, consequating a corresponding rise in the resident population of Assisted Living Facilities (ALFs). This significant increase has highlighted the importance of monitoring residents’ quality of life and reinforcers of enjoyable activity engagement. However, the resident preference assessments currently used for this purpose are outdated and lack specificity. To address this limitation, this study aimed to create an updated measure of ALF residents’ activity engagement reinforcers. The study involved a two-step process. First, 10 subject matter experts in the geriatric field compiled a list of 107 potential reinforcers. This list was then refined into a likert-scale survey of 60 selected items and administered to 612 older adults. Exploratory factor analysis identified five factors (social, traditions, food and activities, family and friends, and outings), and resulted in a 33-item survey. Results of this study provide an effective tool for ALF activity directors and staff to accurately assess ALF residents’ interests and motivators for activity engagement. Clinical implications include connecting at risk ALF residents with individualized program scheduling that may improve their quality of life. This poster reviews the methodology and further results of this survey development.

